# The Effect of Task-Irrelevant Emotional Valence on Limited Attentional Resources During Deception: An ERPs Study

**DOI:** 10.3389/fnins.2021.698877

**Published:** 2021-10-06

**Authors:** Jing Liang, Qian-Nan Ruan, Ke-Ke Fu, Yu-Hsin Chen, Wen-Jing Yan

**Affiliations:** ^1^School of Educational Science, Ludong University, Yantai, China; ^2^Wenzhou Seventh People’s Hospital, Wenzhou, China; ^3^School of Education and Psychology, University of Jinan, Jinan, China; ^4^College of Liberal Arts, Wenzhou-Kean University, Wenzhou, China; ^5^School of Mental Health, Wenzhou Medical University, Wenzhou, China

**Keywords:** deception, emotional valence, task relevance, attentional resources, ERP

## Abstract

Deception is a complex and cognitively draining dyadic process that simultaneously involves cognitive and emotional processes, both of which demand/capture attentional resources. However, few studies have investigated the allocation of attentional resources between cognitive and emotional processes during deception. The current study presented facial expressions of different valences to 36 participants. While an electroencephalogram was recording, they were asked to make either truthful or deceptive gender judgments according to preceding cues. The results showed that deceptive responses induced smaller P300 amplitudes than did truthful responses. Task-irrelevant negative emotional information (TiN) elicited larger P300 amplitudes than did task-irrelevant positive emotional information (TiP). Furthermore, the results showed that TiN elicited larger LPP amplitudes than did TiP in deceptive responses, but not in truthful ones. The results suggested that attentional resources were directed away to deception-related cognitive processes and TiN, but not TiP, was consistently able to compete for and obtain attentional resources during deception. The results indicated that TiN could disrupt with deception and may facilitate deception detection.

## Introduction

Deception is typically defined as a psychological process by which an individual deliberately attempts to convince another to accept as true what the first individual knows to be false, typically to gain benefits or avoid losses for the liar, but sometimes for others (Abe, 2009). Previous studies have shown that deception is a highly complex and cognitively draining dyadic process that simultaneously involves both cognitive and emotional mechanisms (Buller and Burgoon, 1996; Burgoon and Buller, 2015). To successfully deceive another individual, deception simultaneously calls upon numerous cognitive processes to execute, such as response-conflict monitoring, inhibitory control, and/or task switching (Debey et al., 2015; Suchotzki et al., 2015). Furthermore, the deceiver has to constantly monitor his or her target’s demeanor and emotions and control their own throughout the deception process, in order to appear credible and convincing (Vrij et al., 2019). As such, recent deception studies have begun to investigate possible interactions among the cognitive and emotional processes present during deception (Lee et al., 2010; Frank and Svetieva, 2012; Liang et al., 2018). For example, Liang et al. (2018) asked participants to make truthful or deceptive gender judgments when viewing positively and negatively valenced facial expressions. This approach enabled the researchers to investigate the cognitive processes underlying individuals’ truthful and deceptive responses when task-irrelevant emotional information was present. Their results indicated an influence of emotional intensity upon the difference in truthful and deceptive response times, suggesting that the automatic attention-orienting mechanisms of task-irrelevant emotional information were influenced on the cognitive cost of deception. However, it remains unclear how the mind resolves the allocation of finite attentional resources during deception when cognitive and emotional processes are present simultaneously.

A prominent event-related potentials (ERP) in deception tasks is the parietal P3 (also referred to as early P3/late P3/P3b/LPC/LPP according to the time window used in each study) which is a positive deflection typically occurring between 300 and 1,000 ms post-stimulus (Li et al., 2008). The parietal P3 amplitude is selectively sensitive to resources of a perceptual/cognitive nature. Recognition of salient/known stimuli induces larger P3 amplitude than unknown stimuli especially when the knowledge of the stimuli is concealed, as reported in Concealed Information Test studies (Meijer et al., 2014; Leue and Beauducel, 2015). Whereas other deception studies speculate that smaller P3 amplitude reflects attentional resources directed away from the primary task to cognitive processes related to deception (Johnson et al., 2003, 2005; Wu et al., 2009). Specifically, deception researchers generally agree that deception is a cognitively complex process that simultaneously enlists additional mechanisms (i.e., maintenance of truthful information in working memory, inhibition of truthful information, and execution of deceptive responses) beyond the primary task/act of lying itself. As a result, it has been reported through both behavioral (Debey et al., 2012; Suchotzki et al., 2017) and neuroimaging researches (Abe, 2009; Christ et al., 2009) that attentional resources are strained and simultaneously directed across multiple processes during deception. Recent findings on parietal P3 amplitude in tasks requiring deception (Leue et al., 2012; Leue and Beauducel, 2015, 2019) reported individual differences in a deception task for both early P3 (between 300 and 400 ms or 280 and 350 ms post-stimulus) and late P3 (between 400 and 700 ms or 440 and 610 ms post-stimulus). Leue et al. (2012) observed an effect of personality for early P3 amplitude but not for the late P3 amplitude, indicating that early P3 and late P3 amplitudes represent different processes (Leue and Beauducel, 2015).

According to findings of Kayser et al. (2000) who observed larger early and late P3 amplitudes with parietal topographies following emotionally salient stimuli compared to neutral stimuli. It was suggested that early parietal P3 amplitude serve as an indicator of initial affective stimulus salience and should probably not be regarded as a P3a or novelty P3, which is known to have a frontal topography, whereas late parietal P3 amplitude serves as a somatic marker to signal stimulus significance and to guide behavior (Damasio et al., 1991; Kayser et al., 2000; Leue and Beauducel, 2015). Many research found increased LPP amplitude (usually refers to the late P3 amplitude between 400 and 800 ms post-stimulus) following the presentation of emotional rather than neutral stimuli (Hajcak and Olvet, 2008; Hajcak et al., 2010). Furthermore, the LPP amplitudes elicited by negatively valenced pictures were observed to be significantly greater than those elicited by positively valenced pictures, even though both were equally probable, evaluatively extreme, and arousing (Vaish et al., 2008). One study also reported how LPP amplitudes were larger for negatively rather than positively valenced pictures, even when subjects were not asked to explicitly evaluate the valence of the stimulus (Ito and Cacioppo, 2000), demonstrating a negativity bias. In addition, Ferrari et al. (2008) investigated whether LPP amplitudes were simultaneously influenced by task relevance and emotionality, observing the largest LPP amplitudes from task-relevant emotional pictures and the smallest from task-irrelevant neutral pictures. When taken together, these studies suggest that the LPP amplitudes can reflect attentional resources diverted away from the processing of task-relevant stimulus information when both cognitive and emotional processes are simultaneously present in a task that involves a multifaceted stimulus.

However, researchers have yet to explore the division and allocation of finite attentional resources during deception when task-relevant cognitive processes and task-irrelevant emotional information are simultaneously present in a multifaceted stimulus. The present study aims to investigate the above question. Participants were asked to make truthful and deceptive gender judgments about positive and negative facial expressions while behavioral and electroencephalogramic data were recorded. Accordingly, the experiment stimuli included task-relevant information (i.e., gender relevance data such as hair, facial physique, facial features), task-irrelevant positively valenced emotional information (happy facial expression images; from here on referred to as TiP), and task-irrelevant negatively valenced emotional information (angry facial expression images; from here on referred to as TiN).

According to previous studies (Johnson et al., 2003, 2005), we expected significantly reduced parietal P3 amplitudes (for both early and late P3) for deceptive rather than truthful responses indicating that attentional and/or processing resources were being diverted away from processing the stimulus information related to the gender judgment task in order to attend to deception related processes such as maintaining truthful information in working memory, inhibition of truthful information, and execution of deceptive responses which cost more mental effort (Leue and Beauducel, 2019, p. 2). According to negativity bias (Ito and Cacioppo, 2000; Vaish et al., 2008) which indicated negatively valenced emotional information tends to be more salient, we postulated larger parietal P3 amplitudes (both early and late P3) for TiN rather than TiP. Since late P3 amplitude was simultaneously influenced by task relevance and emotionality (Ferrari et al., 2008), we expected the interaction between response (truthful vs. deceptive) and emotion to emerge on late P3 which is required to guide the individual in response selection and decision making by signaling stimulus significance.

## Method

### Participants

Thirty-six undergraduate and graduate students (18 males, average age = 21 years) were recruited. Participants were given a monetary reward for their participation. All were right-handed and had normal or corrected-to-normal vision. All signed informed consents and were approved by the Ethical Committee of Ludong University, in accordance with the Declaration of Helsinki.

### Stimuli and Procedure

Based on the stimulus evaluation results in the study by Liang et al. (2018), 18 happy and 18 angry faces (half female) with intensities above 4.5 from the NimStim and MUG databases were selected for the formal experiment. The average intensity scores were 5.58 (*SD* = 0.57) for the happy faces and 5.76 (*SD* = 0.63) for the angry faces on a 9-point Likert Scale. Independent *t*-tests on the average intensity scores revealed no significant differences between happy and angry faces, *t*(34) = 0.93, *p* = 0.36.

**FIGURE 1:**
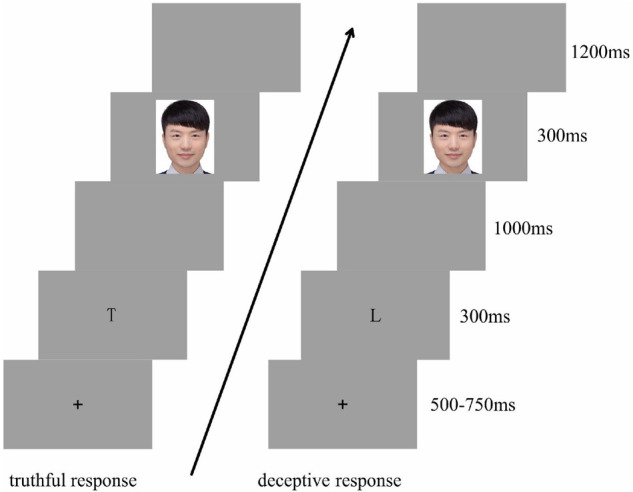
Experiment procedure.

For each trial, following a fixation cross (+) that was visible for 500–750 ms, the cue “T” or “L” appeared for 300 ms. Next, a blank gray screen was presented for 1,000 ms. Then, an image of a facial expression was presented for 300 ms, followed by a blank gray screen for 1,200 ms. The participants judged the gender of the model presented as accurately and quickly as possible, according to the preceding cue. The “F” key was labeled “Male” and the “J” key was labeled “Female.” Participants were instructed to make truthful responses if the cue presented was “T” and deceptive responses if the cue presented was “L.” The “Male” and “Female” keys were counterbalanced among the participants. There were equal numbers of “T” and “L” cues for each block, and all were presented in random order (see Figure 1).

The experiment consisted of three blocks. Each included 72 trials, with each picture repeated twice within a single block. Participants were given as much time as needed to rest before proceeding to the next block.

### Electrophysiological Recording and Analysis

Continuous EEG readings were recorded using 64 electrodes mounted in an elastic cap (Electro-Cap international, Inc.) that was connected to the left mastoid. The data were removed offline and then re-referenced to the average of the left and right mastoids (M1 and M2). The vertical and horizontal electrooculograms were recorded with bipolar channels from sites above and below the midpoint of the left eye and next to the outer canthi of each eye. Mild skin abrasion was performed to reduce electrode impedance below 5 kΩ. The EEG was band-pass filtered from 0.05 to 100 Hz, amplified with a gain of 500, and stored on a computer hard drive at a sample rate of 1,000 Hz (Syn-Amps 4.5, Neuroscan, Inc.).

EOG artifacts from eye blinking and horizontal movement were automatically corrected in all trials using the Scan 4.5 software package. EEGs contaminated with artifacts due to amplifier clipping, bursts of electromyographic activity, or peak-to-peak deflection exceeding ± 75 μV were excluded from the trials. In the present study, the ERP waveforms were time-locked to the time of appearance of the facial expression images. The averaged epoch for the ERPs was 800 ms, including a 100 ms pre-stimulus baseline. Based on previous studies (Leue et al., 2012; Leue and Beauducel, 2015) and inspection of the topographical distribution of the grand-averaged ERP activities, there are two positive peaks in the time interval relevant for the P3 (300–800 ms), one between 300 and 400 ms and another one between 450 and 750 ms after stimulus onset. This indicates that an average across the relevant time window could represent two different components so that both time intervals were used for EEG component quantification. According to Luck (2005), we performed a combined amplitude and latency measurement: In each individual ERP we searched for the most positive segment average of 50 ms within the time window of 300–400 ms after stimulus onset for the early P3 due to its narrow window. The mean amplitude of the late P3 was measured between 450 and 750 ms. The following 15 electrode sites [Fz, F3, F4 (three frontal sites), FCz, FC3, FC4 (three frontal-central sites), Cz, C3, C4 (three central sites), CPz, CP3, CP4 (three central-parietal sites), and Pz, P3, P4 (three parietal sites)] were chosen for the statistical analysis. The amplitude of each ERP component was then submitted in an ANOVA with response type (truth vs. deception), valence (positive vs. negative), and electrode zones (frontal vs. frontal-central vs. central vs. central-parietal vs. parietal) as within-subject variables. ERP amplitudes were averaged over electrodes within a zone.

## Results

### Behavioral Data

**FIGURE 2:**
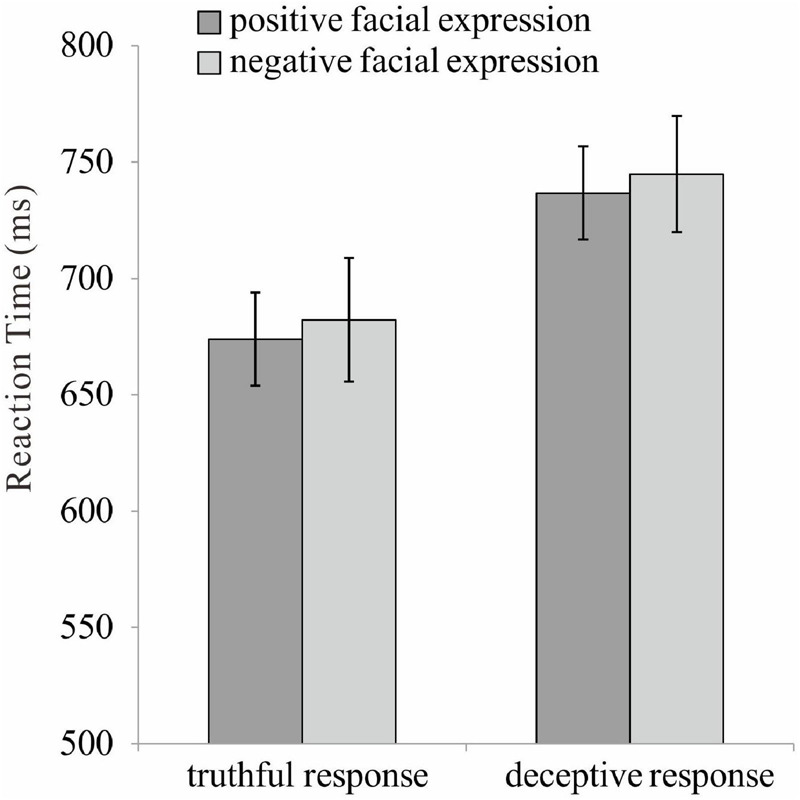
The reaction time for positive and negative facial expression under truthful and deceptive condition.

A series of 2 (valence: positive vs. negative) × 2 (response type: truthful vs. deceptive) repeated ANOVAs were conducted on both accuracy and reaction time. The main effect of response type was found for accuracy, [*F*(1, 35) = 15.33, *p* < 0.001, η^2^*_*p*_* = 0.31], indicating a higher accuracy for truthful (*M* = 0.93, *SE* = 0.01) rather than deceptive responses (*M* = 0.90, *SE* = 0.01). The main effect of response type was found for reaction time, [*F*(1, 35) = 118.10, *p* < 0.001, η^2^*_*p*_* = 0.77], indicating a shorter reaction time for truthful (*M* = 678.12, *SE* = 25.90) rather than deceptive responses (*M* = 740.77, *SE* = 25.74). The main effect of valence was found for reaction time, [*F*(1, 35) = 4.42, *p* = 0.04, η^2^*_*p*_* = 0.11], indicating a shorter reaction time for TiP (*M* = 705.33, *SE* = 25.99) rather than TiN (*M* = 713.56, *SE* = 25.47). The other main and interaction effects were not significant (Figure 2).

### Event-Related Potentials Data

**FIGURE 3:**
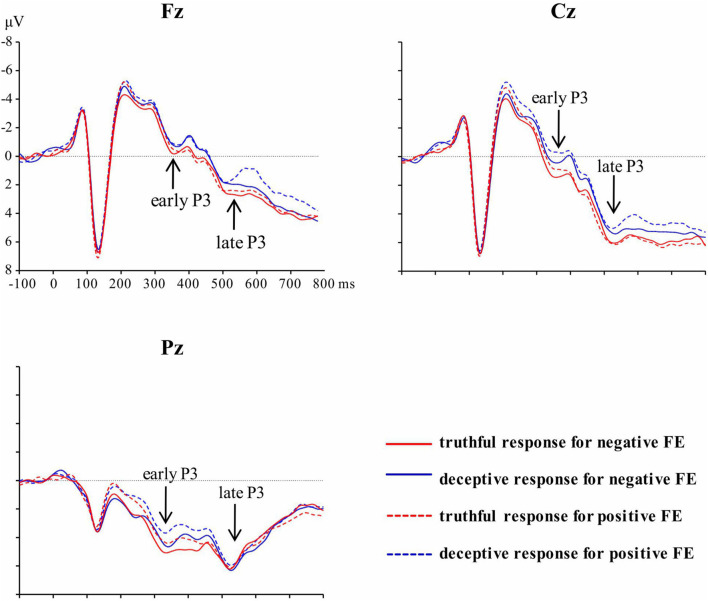
Grand average waveforms at Fz, Cz, and Pz for each of the four experiment conditions.

Figure 3 shows the overall ERP waveforms for the 36 subjects in the four conditions, which were detected using three electrodes on the midline of the scalp. After artifact rejection, the mean numbers of valid trials were 41, 41, 42, and 41 for deceptive responses to negative facial expressions, deceptive responses to positive facial expressions, truthful responses to negative facial expressions, and truthful responses to positive facial expressions, respectively.

#### Early P3 (300–400 ms)

**FIGURE 4:**
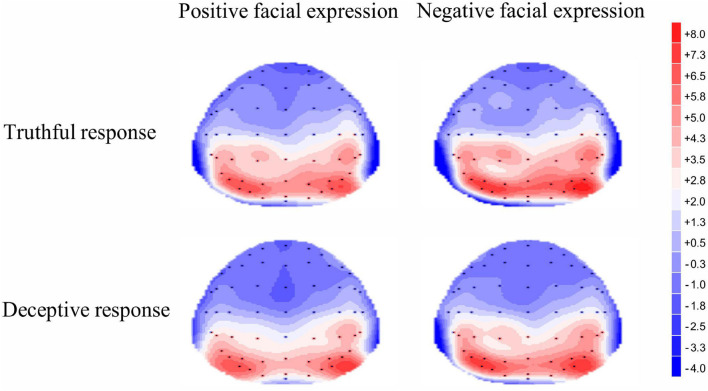
Topographic maps of the mean voltage amplitudes for the four experimental conditions in the 300–400 ms time window.

A significant main effect of response type was revealed for early P3, [*F*(1, 35) = 9.54, *p* = 0.004, η^2^*_*p*_* = 0.21]; the mean amplitude for truthful responses (*M* = 2.94, *SE* = 0.66) was greater than for deceptive responses (*M* = 2.19, *SE* = 0.59). The main effect of valence was significant, [*F*(1, 35) = 4.37, *p* = 0.04, η^2^*_*p*_* = 0.11]; TiN (*M* = 2.74, *SE* = 0.64) elicited a larger early P3 than did TiP (*M* = 2.40, *SE* = 0.59). The main effect of electrode zone was significant, [*F*(4, 140) = 84.48, *p* < 0.001, η^2^*_*p*_* = 0.71], indicating that the amplitude grew greater when moving from the frontal area to the parietal area (0.23, 0.74, 1.99, 4.04, 5.85). The differences among areas are significant except the difference between frontal and frontal-central area. The interactive effect of valence and electrode zone was significant, [*F*(4, 140) = 4.43, *p* = 0.03, η^2^*_*p*_* = 0.11], indicating that the difference between TiN and TiP is significant in the central-parietal and parietal areas (*p* = 0.003, *p* = 0.002), marginally significant in the central area (*p* = 0.06), but not in the frontal and frontal-central areas. The other interactions were not significant. For scalp topographical maps (see Figure 4).

#### Late P3 (450–750 ms)

**FIGURE 5:**
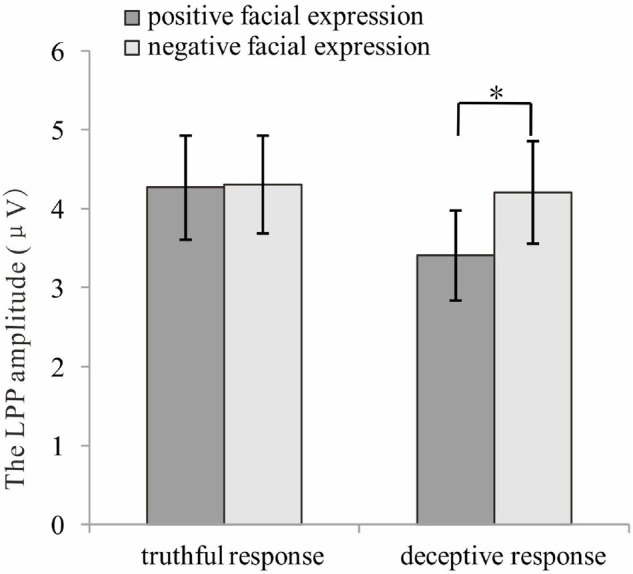
The late P3 amplitudes for truthful and deceptive responses to positive and negative facial expressions. *p < 0.05.

**FIGURE 6:**
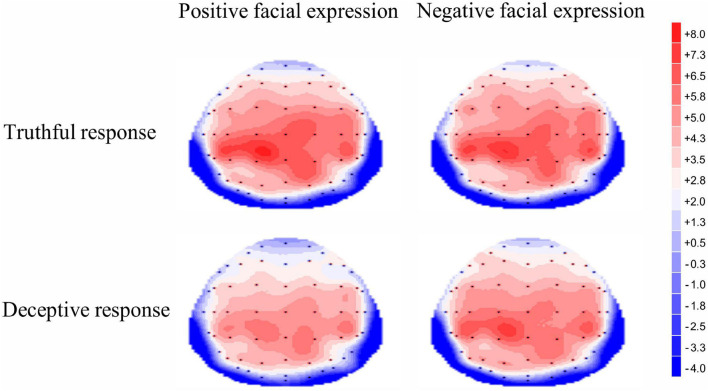
Topographic maps of the mean voltage amplitudes for the four experimental conditions in the 450–750 ms time window.

For the late P3 mean amplitude, there was a significant effect of response type, [*F* (1, 35) = 9.09, *p* = 0.005, η^2^*_*p*_* = 0.21], with the amplitude for truthful responses (*M* = 4.08, *SE* = 0.68) being greater than for deceptive responses (*M* = 3.53, *SE* = 0.66). The main effect of valence was marginally significant, [*F*(1, 35) = 3.49, *p* = 0.07, η^2^*_*p*_* = 009], meaning that TiN (*M* = 3.98, *SE* = 0.68) was associated with a more positive late P3 than was TiP (*M* = 3.63, *SE* = 0.66). The main effect of electrode zone was significant, [*F*(4, 140) = 15.85, *p* < 0.001, η^2^*_*p*_* = 0.31], indicating that the amplitudes in the central, central-parietal and parietal areas (4.51, 5.02, 4.31) were more positive than that in the frontal and frontal-central areas (1.91, 3.26). The interactive effect of response type and valence was significant, [*F*(1, 35) = 4.14, *p* = 0.05, η^2^*_*p*_* = 0.11]. The amplitude for TiN (*M* = 3.84, *SE* = 0.70) was greater than for TiP (*M* = 3.21, *SE* = 0.63) under deceptive conditions, *p* = 0.01. In contrast, the late P3 amplitude for TiN and TiP did not differ under truthful conditions, *p* = 0.80 (see Figure 5). For scalp topographical maps (see Figure 6). The interactive effect of response type and electrode zone was significant, [*F*(4, 140) = 7.15, *p* < 0.001, η^2^*_*p*_* = 0.17], indicating that the amplitude difference between truthful responses and deceptive responses was significant in the frontal, frontal-central, central and central-parietal areas, but not in the parietal areas. The other interactions were not significant.

## Discussion

In the present study, we asked participants to make truthful and deceptive gender judgments of positive and negative facial expressions. An ERP was applied to investigate how individuals resolved and allocated finite attentional resources when a stimulus contained both task-relevant (i.e., gender relevance information) and task-irrelevant emotional information (positively/negatively valenced) during deceptive gender judgments. The results show that deceptive responses induced smaller parietal P3 amplitudes (both early and late P3) than did truthful responses. TiN elicited larger parietal P3 amplitudes (both early and late P3) as compared to TiP. Furthermore, the results reveal that TiN elicited larger late parietal P3 amplitude than did TiP in deceptive but not in truthful responses.

In the present study, significantly reduced early and late parietal P3 amplitudes were observed during deceptive responses in comparison to truthful responses. This result is consistent with previous deception researches suggesting that smaller P3 amplitude (both early and late) reflects attention directed away to other processes present during deception (Johnson et al., 2003, 2005; Wu et al., 2009). In line with recent meta-analysis (Leue and Beauducel, 2019), reduced parietal P3 amplitude in deception can be interpreted in terms of mental effort. Taken together, the results in the present study indicate more mental effort was required when the individuals were cued to make deceptive rather than truthful responses. Significantly larger early and late parietal P3 amplitudes for the TiN trials in comparison to the TiP trials further revealed the influence of task-irrelevant emotional information. Numerous studies have observed differences in parietal P3 amplitude among emotional stimuli of different valences. Specifically, larger parietal P3 amplitude has commonly been reported in negatively valenced stimuli, as compared to positively valenced stimuli (Ito and Cacioppo, 2000; Ferrari et al., 2008; Vaish et al., 2008). In general, previous findings suggest that negatively valenced stimuli have higher saliency than other affectively neutral or even positive stimuli (Ogawa and Suzuki, 2004). Our ERP results mirror those of previous ERP studies, with the results of early and late P3 amplitudes indicating that TiN was more salient compared to TiP.

On top of the main effect for both response type and valence, is the observed interaction between response type and valence for late P3 amplitude. In the present study, larger late parietal P3 amplitude was observed for TiN than for TiP when participants made deceptive responses, whereas no differences in late parietal P3 amplitude were found between TiN and TiP when participants made truthful responses. Previous findings have suggested that late parietal P3/LPP activity indicates the amount of attentional resources being allocated to the processing of a stimulus (Ferrari et al., 2008). In accordance with previous findings, the former result suggests that the amount of attentional resources being allocated to process TiN was greater than for TiP when individuals made deceptive responses. This finding is consistent with the majority of previous findings, which reported that task-irrelevant negative stimuli were more salient and thus demanded more attention (due to processing bias) than task-irrelevant positive stimuli. Yet the latter result suggests otherwise, indicating that the amount of attentional resources allocated to process TiN did not differ from that of TiP when the individuals made truthful responses. Our ERP results imply that when attentional resources are strained (for example, in deceptive responses and when attention needs to be spread simultaneously across numerous cognitive processes), the amount of attentional resources or effort allocated to process TiN is greater than when TiP is present. This indicates that negatively valenced emotional information, despite being task-irrelevant, competed for attentional resources and successfully acquired more attentional resources when those attentional resources were strained during deceptive responses; less attentional resources were allocated to TiP during deceptive responses. In contrast, when attentional resources were not as strained (for example, in truthful response conditions), both TiN and TiP competed for attentional resources and perhaps received equal allocations. Based on the results of the present study, it can be speculated that TiN was able to consistently obtain attentional resources. In contrast, the attentional resources or effort allocated to TiP depended on whether attentional resources were constrained or not.

These results provide a glimpse into how task-irrelevant emotional information influences individuals’ deceptive and truthful responses. Deception detection researches have unveiled numerous findings underscoring the importance of both emotion and cognitive load in deception (Vrij et al., 2012, 2017). The findings of the present study demonstrate how the two equally important processes involved in deception simultaneously compete for and are allocated finite attentional resources. These conclusions have implications for future deception detection research relying on imposed cognitive load to magnify differences between liars and truth-tellers. Numerous studies have reported how additional cognitive loads can effectively aid in deception detection (Frank and Svetieva, 2012; Vrij et al., 2012, 2017). According to that theory and previous findings, additional cognitive load imposed on liars whose cognitive resources have already been partially depleted by the cognitively demanding task of lying, further strain their available resources. As a result, such loading has a particularly debilitative effect on a liar’s attempt to present a plausible, detailed, and convincing depiction of an event.

Based on the findings of the present study, the presence of TiP and TiN should be considered in the process of deception detection. Our results indicate that TiN consistently competed for and exerted strain on attentional resources, regardless of the response type (deceptive vs. truthful). In contrast, the strain TiP imposed on attentional resources in a manner that did not facilitate the goal of overloading and debilitating liars by imposing additional strain. Our results suggest that less strain on attentional resources was imposed when individuals made deceptive responses if TiP was present. Conversely, the strain on attentional resources imposed when individuals made truthful responses did not differ between trials where TiN or TiP was present. These results suggest that the presence of TiP in deception detection may lessen rather than magnify the differences between truth-tellers and liars. However, caution is advised when interpreting results obtained in the present study. Deceptive responses were dictated by the experiment’s instructions, rather than freely undertaken by the participants in our study. Hence, the experiment conditions differed from the real-world high stakes lies considered in previous deception detection research, where the motivation to deceive was strong. A study examining the effect of willingness toward honest and deceptive responses, reported a significant main effect of willingness, observing larger P3 amplitude elicited by self-determined responses compared to forced responses (Wu et al., 2009). Another limitation of this research is the lack of control over possible individual differences in cognitive capacity. Study has reported a relationship between individual differences in executive functions (cognitive capacity) and the accuracy as well as latency of deceptive responses. Results showed that set-shifting and inhibition were directly related to deception accuracy and speed, respectively. However, enhanced underlying working memory skills (both verbal and spatial) were negatively associated with deception speed (Visu-Petra et al., 2012).

## Conclusion

The present study highlighted how emotional and cognitive processes involved in deception simultaneously compete for finite attentional resources. The results show that deceptive responses induced smaller early and late P3 amplitudes than did truthful responses. Additionally, TiN elicited larger early and late P3amplitudes than did TiP. Furthermore, the results reveal that TiN elicited larger late P3 amplitude than did TiP in deceptive but not truthful responses. The results suggest that attentional resources were directed away to deception-related cognitive processes. Moreover, TiN but not TiP, was consistently able to compete for and obtain attentional resources during deception. The results reveal a unique effect of stimulus saliency (in the form of task irrelevant negatively valenced emotional information) during deceptive responses but not truthful responses.

## Data Availability Statement

The raw data supporting the conclusions of this article will be made available by the authors, without undue reservation.

## Ethics Statement

The studies involving human participants were reviewed and approved by the IRB in Ludong University. The patients/participants provided their written informed consent to participate in this study.

## Author Contributions

JL conceived and designed the experiments. W-JY and JL performed the experiments. JL, Y-HC, Q-NR, and K-KF wrote and revised the manuscript. All authors contributed to the article and approved the submitted version.

## Conflict of Interest

The authors declare that the research was conducted in the absence of any commercial or financial relationships that could be construed as a potential conflict of interest.

## Publisher’s Note

All claims expressed in this article are solely those of the authors and do not necessarily represent those of their affiliated organizations, or those of the publisher, the editors and the reviewers. Any product that may be evaluated in this article, or claim that may be made by its manufacturer, is not guaranteed or endorsed by the publisher.
