# Supplementary material for: The Effect of Task-Irrelevant Emotional Valence on Limited Attentional Resources During Deception: An ERPs Study
Source: Front Neurosci. 2021 Oct 6;15:698877. doi: 10.3389/fnins.2021.698877 (PMC8528177; doi:10.3389/fnins.2021.698877)
Supplement: Supplementary file 1 [file Table_1.DOCX]

**N2 (170-300 ms) analysis.**

The mean amplitude of N2 was measured between 450 to 750 ms. The main effect of valence was significant, *F* (1, 35) = 10.58, *p* = 0.003, *η* = 0.23; TiN (*M* = -2.79, *SE* = 0.50) elicited a smaller N2 than did TiP (*M* = -3.38, *SE* = 0.46). The main effect of electrode zone was significant, *F* (4, 140) = 88.81, *p* < 0.001, *η* = 0.72, indicating that the amplitude grew positive when moving from the frontal area to the parietal area (-5.57, -5.41, -3.88, -1.32, 0.87). The differences among areas are significant except the difference between frontal and frontal-central area. The other main effect and interactions were not significant.

The N2 is a negative amplitude deflection over frontal-central scalp sites at around 200-300ms. It is typically conceived of as an index of attentional control (Dennis & Chen, 2009) and N2 amplitude increases have been interpreted as recruitment of cognitive control. Several studies have shown that the amplitude of the N2 is attenuated in response to negative compared to positive stimuli (Olofsson et al., 2008), which means negative stimuli reduced cognitive control relative to positive stimuli, thus supporting the idea that positive stimuli do not need such urgent mobilization of attentional resources as do negative stimuli (Carretié et al., 2004).

P2 (110-330 ms)

We have investigated the EPRs time-locked to the presentation of the “L” or “T”. One subject has been deleted with more bad trials. Result showed that the difference between “T” and “L” cue exists in the time window of 110 ms and 330 ms, which refers to P2. Results showed that the “L” cue elicited a larger P2 (1.25μV) than the “T” cue (0.76μV). The main effect of electrode zone was significant, *F* (4, 136) = 45.21, *p* < 0.001, *η* = 0.57, indicating that the amplitude grew smaller when moving from the frontal area to the parietal area (1.57, 1.46, 1.11, 0.70, 0.21). The differences among areas are significant except the marginal difference between frontal and frontal-central area (*p* = 0.06). The interaction between cue and electrode zone is significant, *F* (4, 136) = 6.96, *p* < 0.001, *η* = 0.17, indicating that difference between “T” and “L” cue is significant in frontal, frontal-central, central and central-parietal areas except the parietal area.

The P2 is usually suggested to play a role in perceptual processing (Chen et al., 2008; Hillyard and Anllo-Vento, 1998). Given the stimulus feature here, the “L” cue could be more arousing and attention-capturing for participants than the “T” cue, which resulted in increased P2.


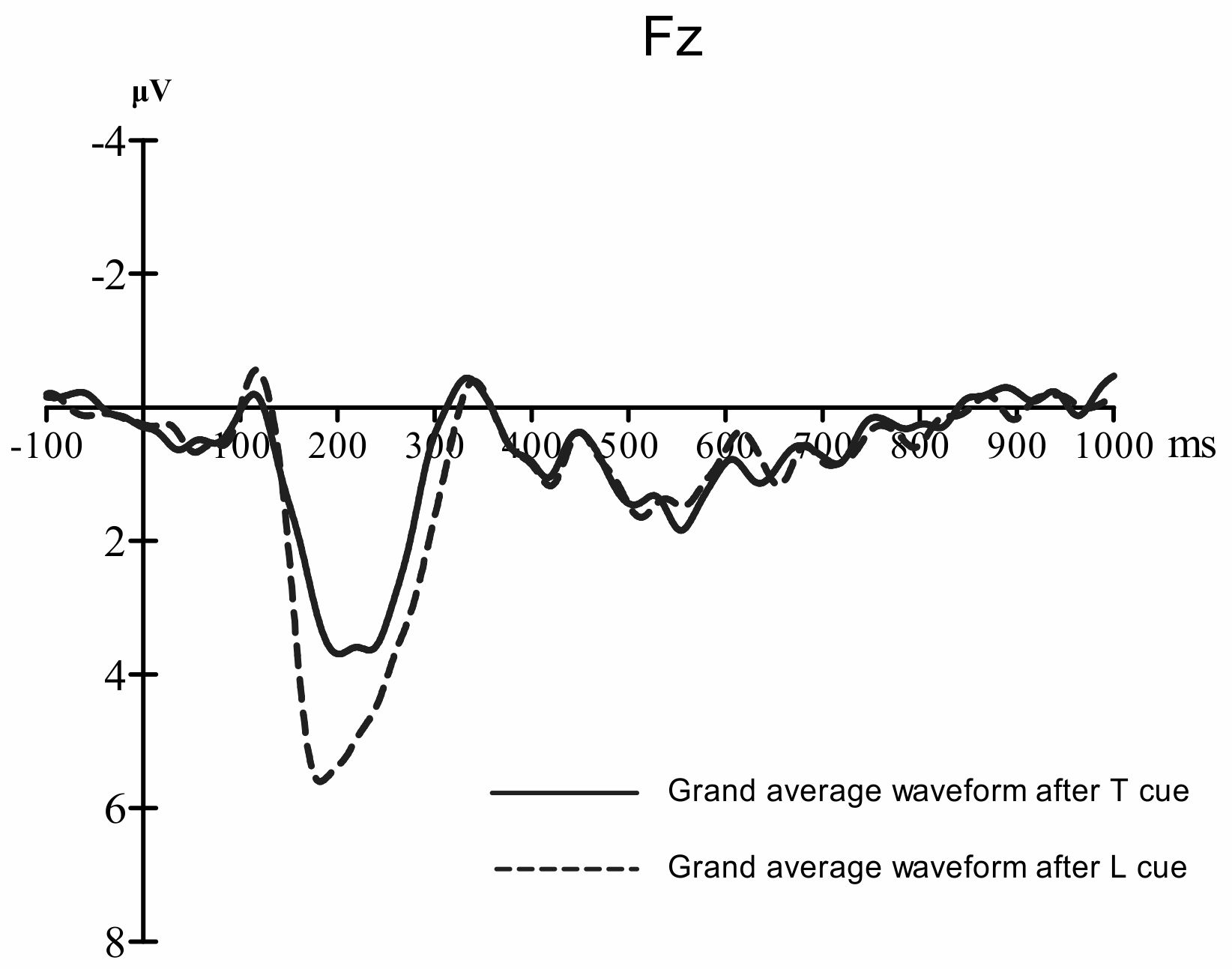


Supplementary Figure 1

References:

Carretié, L., Hinojosa, J. A., Martin-Loeches, M., Mercado, F. and Tapia, M. (2004). Automatic attention to emotional stimuli: Neural correlates, Hum Brain Mapp. 22, 290–299.

Dennis, T. A. and Chen, C. C. (2009). Trait anxiety and conflict monitoring following threat: an ERP study, Psychophysiology. 46, 122-131.

Olofsson, J. K., Nordin, S., Sequeira, H. and Polich, J. (2008). Affective picture processing: an integrative review of ERP findings, Biol psychol. 77, 247-265.

Chen, A., Xu, P., Wang, Q., Luo, Y., Yuan, J., Yao, D. and Li, H. (2008). The timing of cognitive control in partially incongruent categorization, Hum. Brain Mapp. 29, 1028–1039.

Hillyard, S.A. and Anllo-Vento, L. (1998). Event-related brain potentials in the study of visual selective attention, Proc. Natl Acad. Sci. USA 95, 781–787.
